# Detection and comparison of microRNA expression in the serum of Doberman Pinschers with dilated cardiomyopathy and healthy controls

**DOI:** 10.1186/1746-6148-9-12

**Published:** 2013-01-17

**Authors:** Carola Steudemann, Stefan Bauersachs, Karin Weber, Gerhard Wess

**Affiliations:** 1 Clinic of Small Animal Medicine, LMU University of Munich, Veterinaerstrasse 13, Munich 80539, Germany; 2 Laboratory for Functional Genome Analysis (LAFUGA), Gene Center, LMU University of Munich, Feodor-Lynen-Strasse 25, Munich 81377, Germany

**Keywords:** Dobermann Pinscher, Dilated cardiomyopathy, miRNA, Serum, Dog, Microarray

## Abstract

**Background:**

Dilated cardiomyopathy (DCM) is the most common heart disease in Doberman Pinschers. MicroRNAs (miRNAs) are short non-coding RNAs playing important roles in gene regulation. Different miRNA expression patterns have been described for DCM in humans and might represent potential diagnostic markers. There are no studies investigating miRNA expression profiles in canine DCM. The aims of this study were to screen the miRNA expression profile of canine serum using miRNA microarray and to compare expression patterns of a group of Doberman Pinschers with DCM and healthy controls.

**Results:**

Eight Doberman Pinschers were examined by echocardiography and 24-hour-ECG and classified as healthy (n = 4) or suffering from DCM (n = 4). Total RNA was extracted from serum and hybridized on a custom-designed 8x60k miRNA microarray (Agilent) containing probes for 1368 individual miRNAs. Although total RNA concentrations were very low in serum samples, 404 different miRNAs were detectable with sufficient signal intensity on miRNA microarray. 22 miRNAs were differentially expressed in the two groups (p < 0.05 and fold change (FC) > 1.5), but did not reach statistical significance after multiple testing correction (false discovery rate adjusted p > 0.05). Five miRNAs were selected for further analysis using quantitative Real-Time RT-PCR (qPCR) assays. No significant differences were found using specific miRNA qPCR assays (p > 0.05).

**Conclusions:**

Numerous miRNAs can be detected in canine serum. Between healthy and DCM dogs, miRNA expression changes could be detected, but the results did not reach statistical significance most probably due to the small group size. miRNAs are potential new circulating biomarkers in veterinary medicine and should be investigated in larger patient groups and additional canine diseases.

## Background

Dilated cardiomyopathy (DCM) is the most common acquired heart disease in large- and giant-breed dogs. DCM in dogs is therefore an important cause of cardiac morbidity and death [1-3]. Doberman Pinschers are among the most frequently affected breeds. DCM in Doberman Pinschers is inherited and typically shows a unique disease progression with a late onset [1,4-8]. Most dogs with clinical signs of heart disease were diagnosed at an age of 5 to 10 years [9]. Doberman Pinschers at an age of 6 to 8 years showed a prevalence of 43.6% and older dogs a prevalence of 44.1%. Even if disease manifests itself with an increasing age, younger Doberman Pinschers are also affected. The cumulative prevalence of DCM in Doberman Pinschers in Europe was demonstrated to be 58.2% [8].

The natural progression of DCM can be characterized by three stages [1,6,9-11]. Stage I is defined by a morphologically and electrically normal heart. There is no evidence of clinical signs of heart disease in this phase. None of the currently available diagnostic tests can identify this stage. In the second stage (also called “occult stage”), morphologic and/or electrical derangement is presented in the absence of clinical signs of cardiac disease. Dogs typically appear absolutely normal to their owners, despite evidence of abnormalities detectable by cardiac examination. Abnormalities consist of left ventricular enlargement and occurrence of ventricular premature contractions (VPCs). Morphological and electrical abnormalities may coexist or be of predominantly one form at the time of occult stage [1,12-15]. The diagnosis of stage II consists of echocardiographic evaluation of left ventricular dimensions and function and 24-hour ambulatory electrocardiogram (Holter monitoring) evaluating evidence of ventricular arrhythmias. The DCM in Doberman Pinschers usually starts with VPCs, which can lead to sudden cardiac death in about one third of the dogs. Later morphological changes typical for DCM develop, which can be detected by echocardiography. Stage III, the overt stage, is characterized by the presence of clinical signs of heart failure [1,12-15]. For prognostic and therapeutic but also for breeding purposes, it is essential to diagnose DCM before clinical signs develop.

Board-certified cardiologists recommend yearly screening of Doberman Pinschers for DCM by echocardiography and Holter examination, starting at two years of age [4,8]. The combination of echocardiography and Holter monitoring are considered to be the gold standard for the diagnosis of DCM in Doberman Pinschers. But both diagnostic methods have several disadvantages, including high financial costs for the owners, the need for specialized equipment and veterinary training, therefore limited availability and high interobserver variability [16]. The use of circulating biomarkers in the diagnostic approach would offer an attractive alternative because of widespread availability, quantitative nature, potential cost efficiency and minimally invasive sample collection [16]. Therefore various neurohormones and myocardial enzymes as biomarkers of DCM were evaluated [16-20].

MicroRNAs (miRNAs) are short (18 – 25 nucleotide in length) non-coding RNAs which play important roles in post-transcriptional gene regulation. miRNAs regulate gene expression by binding to the 3′-untranslated region of their target mRNA and consequently attenuating protein translation. miRNAs are involved in multiple biological processes [21,22]. It is estimated that more than 60% of human genes are fine-tuned by miRNAs [23].

Multiple studies investigated the potential benefit of circulating miRNAs as noninvasive biomarkers and discovered a remarkable stability of miRNAs in blood [24-26]. The levels of miRNAs in serum are stable, reproducible, and consistent among individuals of the same species [26]. Endogenous circulating miRNAs are stably expressed in human serum, whereas synthetic miRNAs are immediately degraded [24]. However, the exact mechanism how miRNAs enter into the serum and how they are protected from enzymeatic degradation is still unknown [27].

A specific circulating miRNA expression pattern has been described for various pathological conditions. It is presumed that changes in the circulating miRNA expression pattern represent fingerprints for various diseases [28-30] and may even precede detectable changes of standard diagnostic tools [28,31]. Several studies in humans reported circulating miRNAs to be promising biomarkers for cancer [24,30,32,33] and liver injury [28,34].

Various studies evaluated differences of miRNA expression in heart diseases. Several miRNAs were found to be highly enriched in the heart [35-37]. miRNAs were shown to be involved in cardiac development, myocyte growth and arrhythmias following myocardial ischemia [38-43]. Experiments in mice have shown that gain or loss of function of individual miRNAs may elicit different forms of heart disease. Cardiac overexpression of miR-195 in transgenic mice initially induced pathological cardiac growth with disorganization of cardiomyocytes and progressed to an echocardiographically confirmed dilated phenotype at the age of six month that finally resulted in heart failure [44]. Inhibition of miRNA biogenesis revealed DCM, premature lethargy and heart failure in mice [45,46]. Liu et al. demonstrated the essential role of miR-133a in the cardiomyocyte proliferation: miR-133a-1 and miR-133a-2 double-mutant mice developed early large septal defects that led to perinatal death, whereas mice that survived to adulthood succumbed to DCM and heart failure. About 50% of surviving mice died from sudden death [47]. Findings of these studies support the assumption that specific miRNAs play an essential role in the control of cardiac growth and remodeling leading to a dilated phenotype of the heart. In 2007, Ikeda et al. detected different miRNA expression patterns in human heart samples from control patients and patients with DCM, aortic stenosis and ischemic cardiomyopathy (ICM). Eight miRNAs were differentially expressed in ICM and DCM which indicates that each form of heart disease is characterized by a specific miRNA expression profile. Among other miRNAs, let-7c, miR-21, miR-92 and miR-101 were deregulated in DCM diseased tissue in contrast to healthy controls [48]. Later experiments demonstrated miRNA expression differences in heart failure either caused by idiopathic or ischemic DCM and confirmed the aberrant expression of miR-92 in DCM [49]. Levels of miR-208, miR-208b and miR-499 were higher in human endomyocardial samples of DCM patients than in controls. Deregulation of individual miRNA expression was associated with a poor clinical outcome and therefore represents a potential prognostic marker of human DCM [37,50]. Satoh et al. found a significant difference in the expression of miR-21 in DCM diseased tissue as well [50]. An increased risk of DCM was also found in people with genetic polymorphisms in the pre-stages of miR-196a and miR-499 [51]. In humans with chronic heart failure caused by nonischemic DCM, miR-142-3p was found to be upregulated in peripheral blood cells [52].

However, circulating miRNAs are only emerging as potential diagnostic and prognostic biomarkers in cardiovascular disease. Cardiac hypertrophy, myocardial infarction and heart failure were associated with different expression profiles of circulating miRNA [25,31,53-58]. Several studies have also reported the potential of circulating miRNAs as diagnostic, prognostic and therapeutic marker for cardiovascular pathologies like coronary artery disease, stroke, diabetes mellitus or hypertension [55]. With the exception of one study in humans investigating miRNA expression in peripheral blood cells in chronic heart failure caused by different cardiomyopathies (including DCM [52]), studies about circulating miRNA expression patterns in human DCM do not exist to date.

Because of the increasing interest in canine genetics and the benefits of using the domestic dog as a model for human hereditary diseases, investigations on canine miRNAs were initiated [59-64]. In 2007, a search of genetic databases revealed significant conservation of miRNA genes between the domestic dog and the human. Expression levels of seven miRNAs were analyzed in several canine tissues. This was the first study that successfully used quantitative Real-Time RT-PCR (qPCR) assays originally designed for human miRNA detection and showed full conservation of mature sequences of canine and human miRNAs [61]. In 2008, Zhou et al. identified 357 miRNA candidates from the dog genome, 300 of which were orthologous to already characterized human miRNAs [60]. Deregulation of miRNA expression patterns has been reported in various canine neoplastic tissues and muscular dystrophy [59,62-64].

To our knowledge this is the first study about circulating miRNAs in canine DCM. The aim of this study was to investigate whether Doberman Pinschers with DCM and healthy controls display different serum miRNA expression patterns in order to evaluate a possible use of miRNAs as biomarkers in dogs.

## Results

### Patient characteristics

Eight Doberman Pinschers with a mean age of 8.6 years were included in the study and assigned to a DCM (n = 4) and a control group (n = 4). Analysis using the Mann–Whitney test revealed statistically significant differences between the two groups for the parameters ‘ventricular premature contractions’ (VPCs) and ‘left ventricular end-diastolic volume’ (LVEDV), ‘left ventricular end-systolic volume’ (LVESV, p = 0.0143) and ‘ejection fraction’ (EF, p = 0.0286; Table 1).

**Table 1:** Baseline and clinical characteristics of study population in the control and DCM diseased group

	**Control (n = 4)**	**DCM (n = 4)**
**Age (years)**	9.3 ± 2.1	7.9 ± 3.3
**Body weight (kg)**	39.3 ± 8.9	36.6 ± 4.5
**Gender (male/female)**	2/2	3/1
**VPCs/24 hours**	12.75 ± 15.39	2748 ± 4842*
**LVEDV (ml/m**^**2**^**)**	67.68 ± 11.60	120.30 ± 3.85*
**LVESV (ml/m**^**2**^**)**	33.63 ± 7.45	72.50 ± 2.85*
**EF (%)**	49.83 ± 6.37	39.67 ± 3.68*
**Urea (mmol/l)**	6.00 ± 0.68	4.80 ± 1.22
**Creatinine (μmol/l)**	62.25 ± 6.40	65.00 ± 6.58
**cTnI (ng/ml)**	≤ 0.20	0.36 ± 0.04*
**total RNA (ng/μl)**	3.63 ± 0.33	4.23 ± 1.23

Age, body weight, VPCs, LVEDV, LVESV, EF, urea, creatinine, cTnI and total RNA values in mean ± SD. cTnI ≤ 0.2 representing values under the detectable limit. DCM: Dilated cardiomyopathy; VPCs/24 hours: ventricular premature complexes in 24 hours; LVEDV: left ventricular end-diastolic volume; LVESV: left ventricular end-systolic volume; EF: left ventricular ejection fraction; cTnI: cardiac troponin I; RNA: Ribonucleic acid. * p < 0.05 vs control.

### Sample characteristics

None of the dogs had evidence of renal disease with urea and creatinine values in reference ranges. Serum samples were stored at – 80°C for 240 ± 144 days (mean ± SD) prior to analysis. Circulating cardiac troponin I (cTnI) levels showed significant difference between the two groups. Total RNA amounts of the serum samples used did not differ between the two groups (for further baseline characteristics see Table 1).

### miRNA microarray

By employing a highly sensitive and custom-designed 8x60k miRNA microarray (Agilent Technologies, Santa Clara, CA, USA), miRNA expression profiles of canine serum were determined. After subtracting background signals, a total of 404 individual miRNAs could be detected.

**Figure 1:**
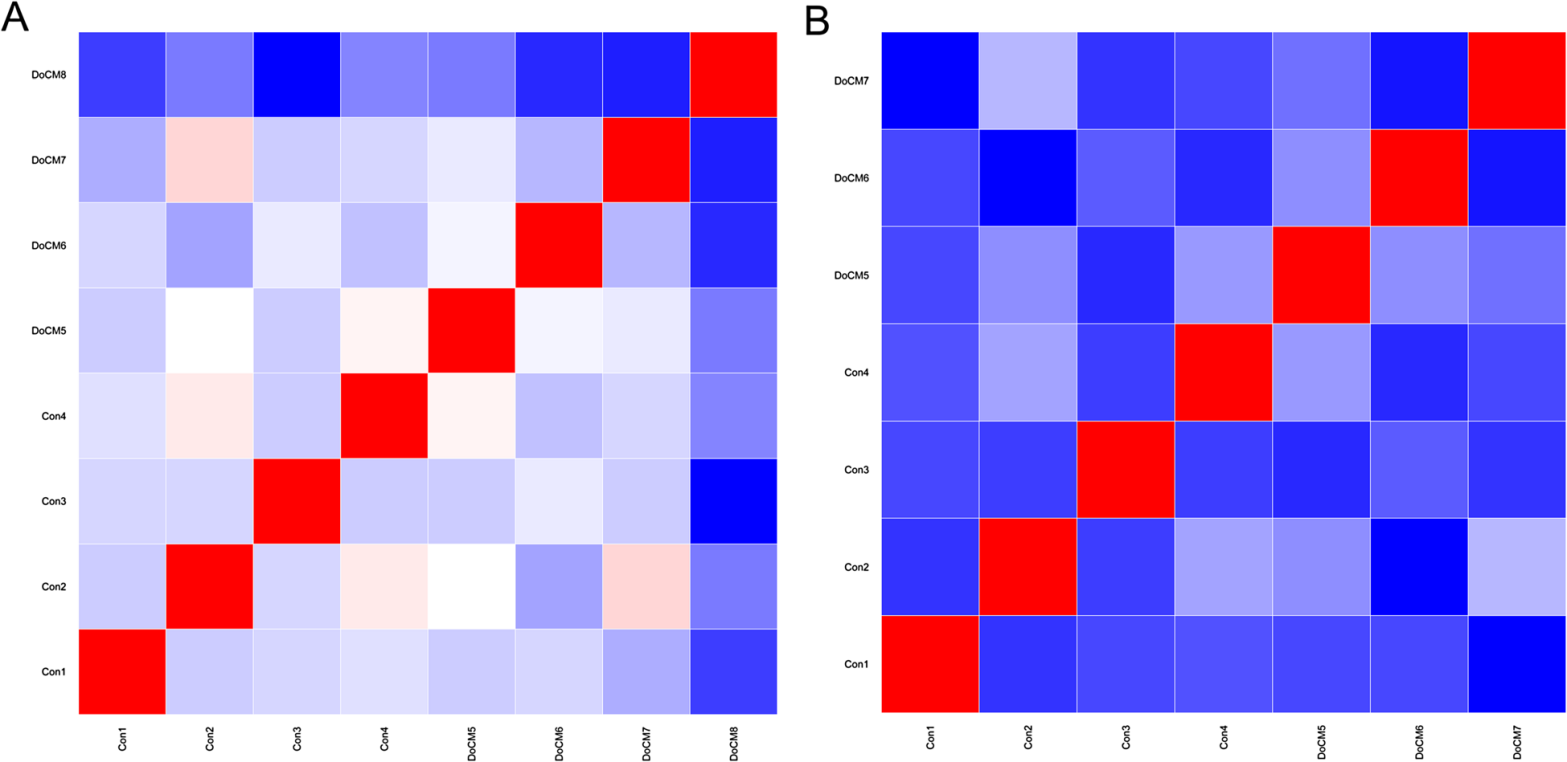
**Matrix of pairwise correlations.** Red coefficient of correlation = 1, blue = lowest values; 1**A**: Correlation matrix showing all samples. Color intensity of DoCM8 is markedly different to other columns indicating strong differences of expression levels compared to other samples. Strong blue colored boxes are illustrating much lower correlation compared to light colored boxes of the other samples. 1**B**: Correlation matrix after exclusion of DoCM8. All boxes are colored similarly indicating uniform variation between all samples. DoCM: DCM in Doberman Pinschers; Con: Control.

**Figure 2:**
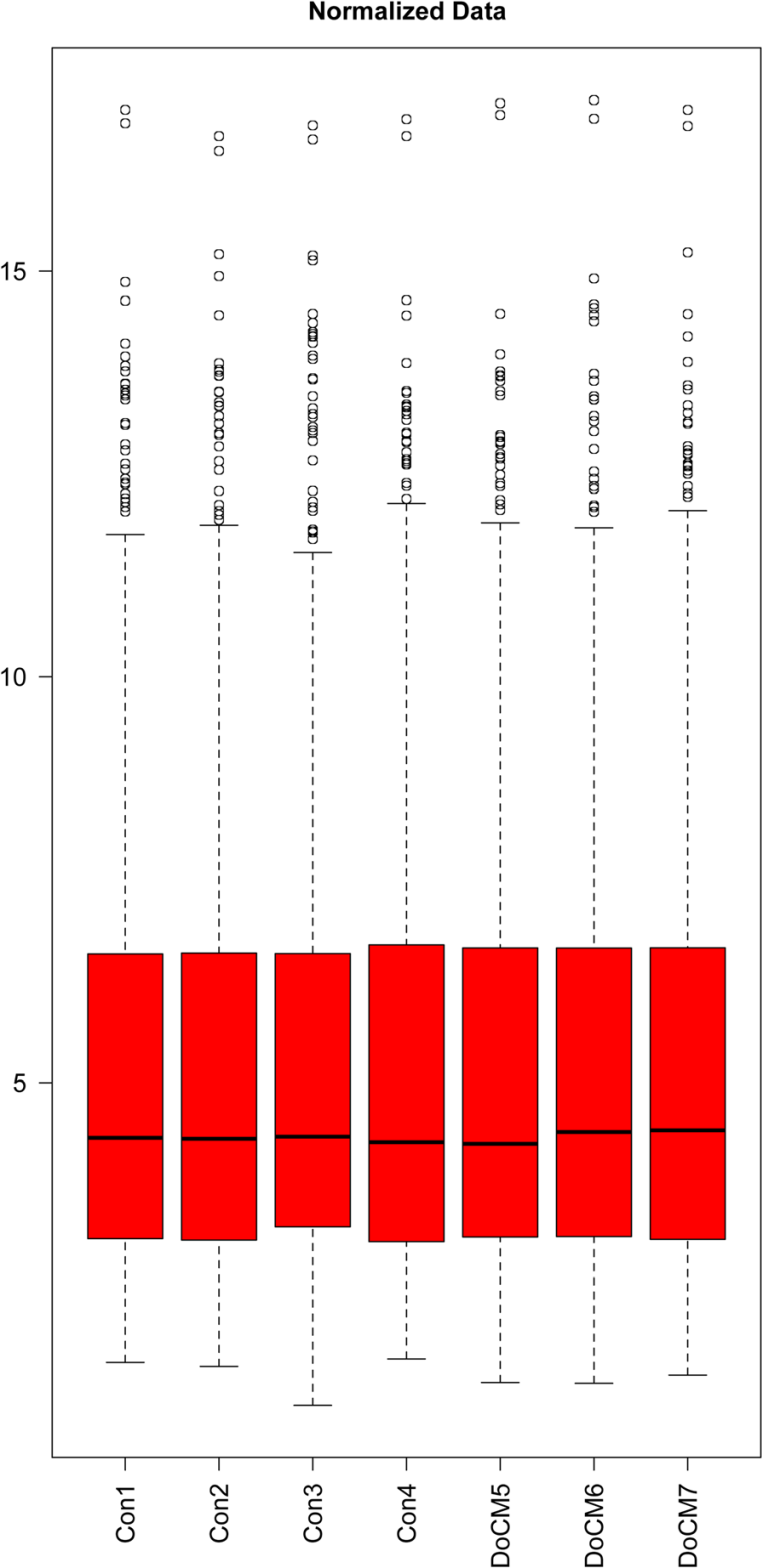
**Whisker-Box Plot showing normalized data after exclusion of DoCM8.** Y-axis: vsn normalized signal intensities (log2 values). The bar near the middle of the box represents the median, the bottom and the top of the box are representing the 25^th^ and the 75^th^ percentile. Circles are displaying outliers. DoCM: DCM in Doberman Pinschers. Con: Control.

One sample of the DCM group was removed from further miRNA microarray analysis because of inconsistency of expression levels compared to other samples (Figures 1 and 2).

**Figure 3:**
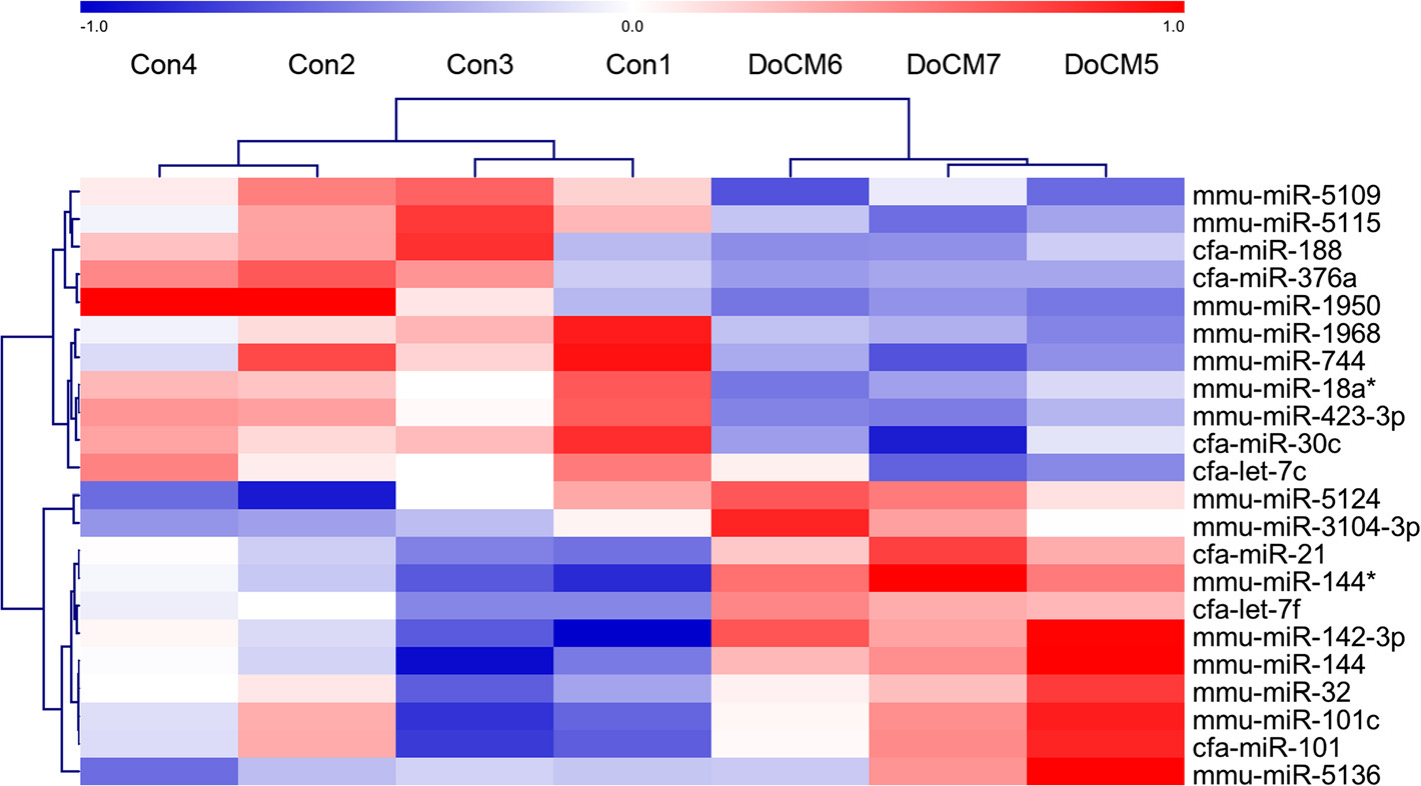
**Hierarchical cluster analysis (HCL support tree) summarizing miRNA expression differences.** Each column represents 1 of 7 samples, each row represents 1 of 22 miRNAs with nominal p-values <0.05. Samples were reproducibly grouped in DoCM and controls after unsupervised hierarchical clustering (MeV 4.7.1, Pearson correlation, HCL support tree, 200 iterations). The different colors of the tree visualize the reliability of branching (black: 100%, grey: 90–100%, blue: 80–90%, green: 70–80%, yellow: 60–70%, ocher: 50–60%, magenta: 0–50%, red: 0% support). Red and blue (rectangles) indicating higher and lower expression levels, respectively, relative to the mean of all samples (log2 mean-centered expression values); DoCM: Dilated Cardiomyopathy in Doberman Pinschers; Con: Control; cfa: canis familiaris, mmu: mus musculus, miR: microRNA.

Expression of 22 miRNAs was different between the two groups (p < 0.05 and fold change (FC) > 1.5, moderated *t*-test, Software “LIMMA”). Expression differences of all miRNAs were relatively small and did not exceed a 2.3-fold change. miR-142-3p showed a 2.18-fold change (p: 0.0042), miR-144* a 2.20-fold change (p: 0.0016), miR-21 a 1.67-fold change (p: 0.0089) and let-7c was negatively 1.53-fold changed (p: 0.0340). Adjusted p-values of all 22 miRNAs after multiple testing correction did not reach statistical significance (false discovery rate adjusted p > 0.05; Table 2). miR-92a was different between the groups but marginally missed the inclusion criteria for the definition of differential expression (cfa-miR-92a: p: 0.0359; FC: - 1.45; mmu-miR-92a: p: 0.0422; FC: - 1.41;). However, miR-92a was considered for further analysis (see qPCR). Hierarchical cluster analysis (HCL support tree) is visualizing differences in miRNA patterns between healthy and diseased dogs. This analysis revealed a clear separation of healthy and diseased samples that was stable with 200 iterations as well as relatively high variation within both groups (Figure 3).

**Table 2:** miRNAs with different expression in the miRNA microarray analysis

**miRNA**	**FC**	**p**	**p adjusted**
**mmu-miR-1950**	−2.14	0.0225	0.6331
**mu-miR-744**	−1.87	0.0117	0.5949
**cfa-miR-30c**	−1.81	0.0141	0.5856
**mmu-miR-5109**	−1.75	0.0185	0.5903
**mmu-miR-423-3p**	−1.74	0.0043	0.5558
**mmu-miR-5115**	−1.66	0.0110	0.5856
**cfa-miR-376a**	−1.62	0.0141	0.5968
**mmu-miR-1968**	−1.58	0.0251	0.6331
**cfa-miR-188**	−1.57	0.0365	0.6405
mmu-miR-18a*	−1.56	0.0159	0.6093
**cfa-let-7c**	−1.53	0.0340	0.6331
**mmu-miR-32**	1.50	0.0476	0.6468
**cfa-let-7f**	1.53	0.0130	0.5968
**mmu-miR-3104-3p**	1.59	0.0226	0.6331
**mmu-miR-5124**	1.65	0.0468	0.6399
**cfa-miR-21**	1.67	0.0089	0.5856
**cfa-miR-101**	1.68	0.0402	0.6331
**mmu-miR-101c**	1.70	0.0394	0.6331
**mmu-miR-5136**	1.82	0.0409	0.6331
**mmu-miR-144**	2.02	0.0067	0.5640
**mmu-miR-142-3p**	2.18	0.0042	0.5640
mmu-miR-144*	2.20	0.0016	0.5476

p < 0.05, FC > 1.5; p adjusted with FDR. FC: fold change, FDR: false discovery rate, cfa: canis familiaris, mmu: mus musculus, miR: microRNA.

#### qPCR

**Figure 4:**
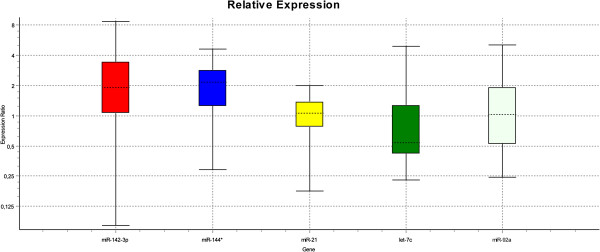
**Whisker-Box Plots showing expression ratios of miRNAs in the qPCR.** Expression ratios in the diseased group were compared to healthy control and normalized to the control assays hs_RNU6B and hs_RNU1A. miR-142-3p and miR-144* were up regulated about two-fold in the DCM group compared to the control group, while let-7c appears to be slightly down regulated. The long whiskers represent the high interindividual variance of expression. Expression ratio of miR-92a and miR-21 is nearly 1. No statistically significant differences between the groups are evident. The dotted line in the box are representing the sample median, the bottom and the top of the box are representing the 25^th^ and 75^th^ percentile and the whiskers are displaying the outer 50% of observations. miR: microRNA.

For further analysis, we selected five miRNAs which have been earlier mentioned in literature as being involved in cardiovascular pathology and which also showed a trend for differential expression in the microarray (miR-142-3p, miR-144*, miR-21, let-7c and miR-92a). Five target miRNAs and two endogenous controls were measured by qPCR in the same serum samples used previously for the microarray. The endogenous controls hs_RNU6B_2 and hs_RNU1A_1 did not show a difference in average Δ Δ threshold cycle (Ct) between the groups (hs_RNU6B_2: 27.04 ± 0.62 vs 27.3 ± 0.75; hs_RNU1A_1: 15.72 ± 0.57 vs 15.27 ± 0.94; mean ± SD). miR-142-3p and miR-144* showed a trend of upregulation in the diseased group, whereas let-7c was slightly downregulated in DCM dogs. Differences between the groups remained very small. Results showed a high interindividual variance of expression. While the miRNA microarray showed small differences in expression of miR-21 and miR-92a, qPCR revealed almost no differences of these miRNAs between the two groups (Figure 4). With p-values > 0.05 for all tested target assays, qPCR revealed no statistically significant difference between diseased and healthy dogs (mmu-miR-142-3p: p = 0.771; mmu-miR-144*: p = 0.421; cfa-let-7c: p = 0.634; cfa-miR-21: p = 0.940; cfa-miR-92a: p = 0.873).

## Discussion

To date, the role of miRNAs in human DCM has only been studied using cardiac tissue samples [44,48,50,65,66]. These samples are difficult to obtain and studies on circulating serum miRNAs present a much less invasive approach, although the origin and circumstances of miRNA release into the circulation are currently unknown. Myocardium specific miRNAs have been found to be significantly increased in the serum as soon as one hour after myocardial infarction in humans and mice [31,67]. The spontaneous disease model of canine DCM in the Doberman Pinschers may represent a useful target for translational research. We limited the sample volume to approximately 2 ml of whole blood to complete both microarray and qPCR analysis, which can safely be acquired even from small veterinary patients without adverse effects.

According to manufacturer’s protocols, most microarray systems require a minimal amount of 100 ng total RNA of each sample to be examined. We used in average 117 ng (84–162 ng) total RNA per sample and more than 400 miRNAs showed a sufficient intensity for evaluation. However, higher RNA concentrations may have resulted in more miRNAs to be detected. A standard normalization for miRNA expression analysis of serum samples using microarrays has not been established so far, but normalization of serum volume is reported to be the best method to determine quantity of miRNAs in blood using qPCR [67]. Using the same amount of total RNA in each sample could have produced less interindividual variation in microarray and PCR analysis and should be investigated in further experiments.

We excluded one sample of the diseased group from further analysis of microarray data since miRNA expression levels were extremely different to others even after normalization. This sample was taken from a young dog and had the longest storage time. Total RNA yield from this sample was very low compared to the other samples, whereas signals of miRNAs were intensified in this sample. Patient age is described not to influence miRNA levels in plasma [25] and miRNAs are claimed to be robust against long freezing time [24,68]. Reasons for the difference of this sample remain unexplained.

Since the miRNA database contains more murine and human miRNAs than canine sequences we chose to use both canine and murine targets for our microarray. Sequences have been found to be highly conserved among species with many mature miRNA sequences displaying 100% homology [36,61,63]. The microarray results confirm that canine miRNAs hybridize with multiple murine targets, which facilitates studies using veterinary samples. The phylogenetic relationship of mouse, dog and human is a matter of debate and not unequivocally determined [69,70]. Homology in coding genes might not reflect the situation of noncoding DNA, some miRNA clusters have been found to be unique to primates. In a pilot study we tested mouse serum as positive controls for qPCR detection of several miRNAs vs canine serum and detected the same PCR products in both samples using murine assays. Notably for miRNA studies of cardiac tissue, more information is available for murine samples, including studies on knockout mice. Additionally, a recent analysis of the heart transcriptome shows a higher similarity between dog and mouse than between dog and human [71]. As stated in the manuscript it cannot be ruled out that some murine probes on the array do not match the canine miRNAs, but this would also be the case for human probes. Whether human or murine miRNAs as a whole are more closely related to dog miRNAs and which one would be the ‘better’ model so far remains an open question.

In our study, the differences in miRNA expression between the two groups were not large and consistent enough to reach statistical significance when the results were adjusted for multiple testing. Although single miRNAs were not statistically significant between the two groups, hierarchical cluster analysis of several miRNAs showed a clear separation of diseased and control samples and enabled a reliable differentiation between healthy and diseased dogs. Therefore, a combined expression analysis of several miRNAs may be a new diagnostic tool for the detection of DCM in Doberman Pinschers and should be further investigated in future studies. This study with its comparatively small patient cohort should be considered as a pilot study requiring confirmation and validation of results in larger sample groups.

A microarray study comparing miRNA expression in heart tissue samples from several human heart diseases revealed let-7c to be significantly upregulated and miR-101 to be significantly downregulated in human DCM, although the changes were less than two-fold [48]. In contrast, our study detected a downregulation of let-7c and an upregulation of miR-101, but the changes were quite subtle.

The let-7 family has been shown to be one of the most abundant miRNAs in murine heart tissue [46]. Recently, a decrease of myocardial let-7i was associated with severity of left ventricular dysfunction and poor clinical outcome in human DCM patients and therefore supposed to be a potential prognostic marker [50]. Let-7i was reported to target for toll-like receptor 4 (TLR4) *in vitro*, a critical transcription factor in the myocardium of DCM in humans [72,73]. In the presented study of canine DCM of Doberman Pinschers, expression changes of let-7i were not evident, but it is not known to date whether TLR4 is a critical transcription factor in canine DCM.

miR-101 has been addressed in several studies as a potent tumor suppressor and inhibitor of autophagy and is obviously expressed in many different tissues [74,75]. A specific regulating effect on the cardiovascular system has not been detected to date.

miR-21 is universally expressed in various organs like spleen, small intestine, colon and the heart [36]. Several studies demonstrated the overexpression of miR-21 in tumor tissues [76,77]. It is also highly expressed in vascular smooth muscle cells, endothelial cells, cardiomyocytes and cardiac fibroblasts [66,78-81]. Deregulated expression of miR-21 has been described for proliferative vascular disease, cardiac hypertrophy, heart failure and ischemic heart disease [44,78,80,82-85]. Myocardial degeneration with cardiac fibrosis and myointimal hyperplasia of the coronary vessels are common histologic findings in the DCM of Doberman Pinschers [6,10,86,87]. miR-21 is upregulated during cardiac remodeling in response to cardiac stress and leads to fibroblast proliferation and fibrosis in humans [66,88]. In the Doberman Pinschers suffering from DCM, miR-21 appeared to be slightly upregulated in the microarray, but the trend could not be confirmed using qPCR assays. In human DCM heart tissue samples, one study group found a subtle and non-significant downregulation of miR-21 [48], while another study claimed miR-21 to be significantly upregulated about two-fold compared to healthy controls [50]. Recently, experimental studies demonstrated an increase of miR-21 expression in right ventricular cells induced by drug therapy. Due to the increase of miR-21, right ventricular function during right heart failure improved as well [89].

In miRNA microarray, miR-92a was slightly downregulated in DCM diseased Doberman Pinschers, but this could not be confirmed using the qPCR assay. As mentioned above, a related miRNA, miR-92, was shown to be downregulated in human DCM tissues [48,49]. Overexpression of miR-92a inhibits angiogenesis in ischemic myocardial tissues in mice and is involved in the control of cardiomyocyte survival. Experiments of external inhibition of miR-92a led to improved left ventricular function [90]. Since all of the diseased dogs received positive inotropic therapy (Pimobendan), this may influence miR-92a expression. So far, very little is known about the effects of cardiovascular therapy on miRNA expression.

For miR-142-3p and miR-144*, we found a trend for upregulation in DCM diseased dogs both in the microarrays and the qPCR results, although interindividual variation was very high. miR-144 was also upregulated in the microarray analysis. miR-144 and miR-144* are the two strands of the double stranded precursor miRNA (mir-144). miR-144 is known to be expressed in a cluster with miR-451. The miR-144/451 promoter is activated by GATA-4, a critical transcription factor in the heart. Both partners of the cluster are supposed to confer protection against stimulated ischemia/reperfusion-induced cardiomyocyte death and may therefore represent possible therapeutic agents for the treatment of ischemic heart disease in humans [91]. The left ventricular dilation, which is a characteristic sign of DCM, leads to myocardial stress. The upregulation of miR-144 might be a stress response of affected myocytes and an attempt to protect the cells against this stimulus. Recruitment of a larger canine patient cohort may allow further validation of these findings.

In accordance with our findings, miR-142-3p has been shown previously to be upregulated in human patients with chronic heart failure caused by nonischemic DCM [52]. This miRNA is one of five negatively regulating cardiac hypertrophy in recent experimental models. Therefore, miR-142-3p is currently discussed as a part of potential therapeutic targets for the treatment of cardiac hypertrophy [92].

The mechanism and exact circumstances of miRNA release into the serum are not fully understood to date [27]. Protected by protein-bound complexes or incorporated into lipid carriers, miRNAs are resistant to RNases in circulation. It is estimated that about 80% of circulating miRNAs are secreted in protein complexes. The other part of circulating miRNAs is released in membrane-bound vesicles (apoptotic bodies, microvesicles, exosomes) [93-97]. It is suspected that the release of miRNAs into the circulation occurs selectively and the existence of a specific packaging mechanism is therefore suggested. For example, in a metastatic gastric cancer cell line, members of the let-7 family are selectively released into the circulation [98]. Similarly, different human cell lines secrete certain miRNAs upon serum deprivation as part of a stress response [93]. The correlation between circulating and tissue miRNA expressions is still controversial. Diseased cells might not be the only origin of changes in circulating miRNAs. It is also possible that currently unknown signals, e.g. stress signals, are sent from the diseased tissue to other organs in the body and cause a specific release of miRNAs into the circulation. Several experimental studies have suggested that circulating miRNAs can be transferred to target cells and regulate gene expression [99,100]. Circulating miRNA expression patterns might therefore differ from expression patterns extracted from the diseased organ itself. This could explain the discrepancies of circulating miRNA expression patterns found in this study to cellular miRNA expression patterns found in other studies.

All dogs in the DCM group received a background therapy consisting of ACE-Inhibitors and Pimobendan and either Sotalol or Amiodarone as antiarrhythmic therapy. It is possible that medication did not only improve cardiac abnormalities but also influenced miRNA expression changes. The elevated cTnI levels of diseased dogs in this study reflect ongoing myocardial injury. Studies comparing circulating miRNA expression in different disease stages of DCM are needed to verify temporal changes in the course of this disease. No studies are published about a possible influence of the drugs used to treat the dogs in this study on the miRNA expression. For the purpose of this study only dogs with unequivocal echocardiographic in addition to unequivocal electrocardiographic changes were selected. Therefore, only patients in advanced disease stages were selected and it would have been unethical not to treat those dogs. All dogs received the same background therapy (Pimobendan and ACE-inhibitors), but antiarrhythmic therapy was individualized [15,101]. Antiarrhythmic therapy was adjusted according to the patient’s individual electrocardiographic changes and was therefore not completely homogenous. In some cases, antiarrhythmic therapy was changed in later visits (after serum sampling) because of inadequate response to the administered medication (change from Sotalol to Amiodarone, combination of Amiodarone and Mexiletin or combination of Sotalol and Mexiletin).

## Conclusions

In conclusion, this study demonstrates that miRNA expression profiling is technically feasible in a clinical setting, revealing its potential as a possible new diagnostic approach in veterinary medicine. Standardized protocols for normalization and larger group sizes for different diseases are needed to confirm whether circulating miRNAs may serve as biomarkers in dogs in the future and whether canine disease models display changes similar to human heart diseases.

## Methods

### Ethic statement

The dogs in this study were examined during preventive diagnostic procedures with the written consent of their owners (to participate in a study in which remaining blood samples not used for necessary diagnostic procedures are stored and used for genetic, miRNA and other diagnostic research procedures). All local regulations (Germany) were strictly observed. The study was approved by the University of Munich Committee on Research Ethics. There is no permit number as this study is not based on an invasive animal experiment. The data were obtained during routine diagnostic procedures. As the data are from client-owned dogs that underwent normal veterinary exams, there was no "animal experiment" according to the legal definitions in Germany. Nonetheless, the study was reviewed and approved by the local ethics review boards.

### Study population

Eight purebred Doberman Pinschers were retrospectively selected from a large study cohort of the Ludwig-Maximilians-Universität München (LMU Munich) with a longitudinal study design starting in 2004. Each examination included a questionnaire completed by the owner, recording of body weight, physical examination, short-time electrocardiogram (ECG), Holter monitoring and echocardiography. Echocardiography included M-Mode and Simpson’s method of disc measurements as well as color and spectral Doppler measurements. Venous blood was collected from each patient for screening of serum parameters and the samples were stored for further analysis with the owner’s consent. We included only purebred Doberman Pinschers without evidence of systemic disease. Exclusion criteria were evidence of concomitant congenital heart diseases or mitral valvular disease in echocardiography. We retrospectively chose eight dogs which met selection criteria of groups as defined below.

#### ECG

Dogs were positioned in right lateral recumbency. ECG was performed according to standard technique with a twelve channel ECG machine (Schiller Cardiovit AT-10, SCHILLER Medizintechnik GmbH, Germany). Electrical activity was recorded for about 30 seconds and printed for evaluation of any electrical abnormalities. A concurrent ECG was recorded during echocardiography without getting printed.

#### Echocardiography

Echocardiography was performed without sedation in right and following left lateral positions. Examinations were performed with a high frame rate ultrasound system with 2.0/4.3 MHz probes and simultaneous ECG recordings (Vivid 7 dimension, General Electric Medical System, Waukesha, WI). Simpson’s method of disc measurements were recorded in right parasternal long-axis and left apical four chamber views. All valves were examined by color Doppler technique. Spectral Doppler was used for velocity measurement over the aortic and pulmonic valves and had to reveal no evidence of abnormalities.

#### Holter examinations

Holter monitoring was performed following echocardiography. The electrical activity was recorded for 24 hours in the normal environment of the dogs without clinical habitation. Monitor was removed after 24 hours and commercially available Holter analysis systems were used for analysis of the digitally recorded ECGs (Custo tera, Arcon Systems GmbH, Starnberg, Germany; Amedtech ECGpro Holter software, EP 810 digital recorder, Medizintechnik Aue GmbH, Aue, Germany). Manual adjustments and verification of accuracy of systemically detected arrhythmias were performed by veterinarians with experience in Holter analysis. Total numbers of VPCs were tabulated.

#### Blood preparation

Blood (5 ml) was drawn from jugular vein of each patient and allowed to clot at room temperature for at least 20 minutes. The sample was centrifuged at 600 g RCF for five minutes at room temperature and the serum was transferred to 0.5 ml polypropylene tubes (Eppendorf, Hamburg, Germany) and stored at - 80°C until RNA isolation.

Four dogs each were grouped into two different groups according to the results of the electrocardiographic and echocardiographic examination:

*Control group:* Dogs in this group had an age of seven years or older and no clinical signs of systemic or cardiac disease. Renal parameters had to be in the reference range (Urea: 3.3 – 8.3 mmol/l, Creatinine: 31.8 – 117 μmol/l) and cTnI levels had to be ≤ 0.2 ng/ml. There were no electrical abnormalities in the ECG: all amplitudes, duration of PQ-interval and mean electrical axis were in normal ranges, dogs had < 50 VPCs in the Holter examination. Echocardiographic measurements were considered to be normal. This includes no or only trivial insufficiencies of the valves. Simpson’s method of disc was used for calculation of the left ventricular end-diastolic (LVEDV) and end-systolic (LVESV) volume normalized to the body surface area (BSA). LVEDV/BSA ≤ 100 ml/m^2^ and LVESV/BSA ≤ 55 ml/m^2^ were considered as normal [102]. The ratio of the left atrium to the aorta (LA/Ao) was 1.5 or less. We are in frequent communication by phone with all owners. We perform yearly screenings to monitor healthy dogs and to detect even early changes. All healthy dogs are alive and healthy at the time of reviewing this paper. We already performed follow-up evaluations in 2012 and could not detect any evidence of DCM in these dogs.

*DCM group (occult stage):* Dogs in this group had no clinical signs of systemic disease. Owners did not observe any events of syncope or exercise intolerance. Clinical signs of cardiovascular abnormalities (pale mucosal color, weak pulse, pulse deficit or arrhythmic pulse, heart murmur) were acceptable. Renal parameters had to be in reference range and circulating cTnI levels were elevated (> 0.2 ng/ml). Electrical and morphological abnormalities existed in the current visit or in one of the earlier visits (partly normal under therapy in the current visit): Dogs had > 100 VPCs in the Holter examination and echocardiographic changes including LVEDV/BSA > 100 ml/m^2^ and LVESV/BSA > 55 ml/m^2^[102]. LA/Ao had to be 1.5 or less. We performed frequent control examinations every 1 – 6 months to monitor development of disease. After the diagnosis of DCM, we performed 4 – 6 follow-up examinations of dogs in this group. The time course of electrocardiographic and echocardiographic changes is listed in Table 3. Due to the severe existing changes, all dogs received therapy at the time of serum sampling. Dosing of drugs was standardized. Further characteristics of diseased dogs are listed in Table 4.

**Table 3:** Time course of electrocardiographic and echocardiographic changes of dogs with DCM starting from time of diagnosis

**Sample name**	**Number of visits**	**VPCs/24 hours**	**Maximal velocity of VPCs (bpm)**	**LVEDV (ml/m**^**2**^**)**	**LVESV (ml/m**^**2**^**)**	**EF (%)**
**DoCM 5**	1^st^ visit	254	240	119.20	65.80	44.67
	2^nd^ visit	107	203	94.60	56.70	40.08
	3^rd^ visit*	161	200	116.80	73.10	37.41
	4^th^ visit	199	214	110.90	62.40	43.72
**DoCM 6**	1^st^ visit	13641	300	105.70	63.40	39.97
	2^nd^ visit	10355	319	124.20	79.40	36.06
	3^rd^ visit*	10005	300	122.50	68.40	44.21
	4^th^ visit	14	196	112.30	77.60	30.93
	5^th^ visit	22	230	132.50	78.20	41.01
	6^th^ visit	24	300	142.50	94.50	33.73
**DoCM 7**	1^st^ visit	1940	230	112.70	72.90	35.31
	2^nd^ visit	2478	275	121.70	82.40	32.31
	3^rd^ visit	17	273	116.40	79.90	31.40
	4^th^ visit*	616	180	117.30	75.00	36.04
	5^th^ visit	3010	250	151.20	108.50	28.24
	6^th^ visit	135	300	143.70	123.30	14.19
**DoCM 8**	1^st^ visit	302	300	116.00	72.30	37.68
	2^nd^ visit	9	265	109.90	59.80	45.62
	3^rd^ visit*	209	202	124.60	73.50	41.01
	4^th^ visit	7295	300	99.00	50.50	48.93

DCM: Dilated cardiomyopathy, DoCM: Dilated Cardiomyopathy in Doberman Pinschers, VPCs: Ventricular premature complexes, VPCs/24 hours: ventricular premature complexes in 24 hours; bpm: beats per minute; LVEDV: left ventricular end-diastolic volume; LVESV: left ventricular end-systolic volume; EF: left ventricular ejection fraction, *: visit of serum sampling for microRNA analysis.

**Table 4:** Further characteristics of DCM diseased dogs

**Sample name**	**Age of diagnosis of DCM (years)**	**Age at the date of serum sampling (years)**	**Time of treatment prior to serum sampling (days)**	**Drug therapy administered until the time of serum sampling**	**Outcome**	**Age at the time of death (years)**
**DoCM 5**	11.2	11.5	140	Pimobendan, ACE Inhibitor, Sotalol	Other systemic disease (gastric volvulus)	11.9
**DoCM 6**	7.5	7.8	119	Pimobendan, ACE Inhibitor, Amiodarone	Sudden cardiac death	8.6
**DoCM 7**	7.4	8.5	397	Pimobendan, ACE Inhibitor, Sotalol	Euthanasia because of non-responsive congestive heart failure	9.2
**DoCM 8**	3.0	3.6	321	Pimobendan, ACE Inhibitor, Amiodarone*	Euthanasia because of non-responsive congestive heart failure	4.3

DoCM: Dilated Cardiomyopathy in Doberman Pinschers. ACE: Angiotensin Converting Enzyme. *: The medication was not consistently administered because of incompliance of the owner.

### RNA extraction

Total RNA was isolated using the miRNeasy Mini Kit (QIAGEN, Valencia, CA, USA) according to the supplementary protocol “Purification of total RNA, including small RNAs, from serum or plasma using the miRNeasy Mini Kit”. Briefly, 400 μl serum were mixed with 2 ml of QIAzol Lysis Reagent and 400 μl Chloroform. The sample was centrifuged and the upper aqueous phase was transferred to a collection tube and mixed with 975 μl 100% ethanol. The mixture was applied to RNeasy Mini spin columns and purified according to the protocol. Total RNA was eluted using 50 μl of RNAse free water. RNA concentrations were determined using a NanoDrop ND-1000 spectrophotometer (NanoDrop Technologies, Wilmington, DE, USA). Samples were stored at -80°C.

### miRNA microarray

The miRNA Microarray System with miRNA Complete Labeling and hybridization Kit (Agilent Technologies) was used according to the manufacturer′s recommended protocol. The Agilent microRNA Spike-In Kit was used for in-process control to measure labeling and hybridization efficiency. The RNA samples were dried in a Vacuum Contractor (Bachofer Plumbing Heating & Air Conditioning, Salina, KA, USA) for one hour. All samples were dephosphorylated by incubation with calf intestinal phosphatase at 37°C in a heat block for 30 minutes and incubated in 100% DMSO at 100°C for seven minutes for denaturation. All samples were labeled with pCp-Cy3 using T4 ligase by incubation at 16°C for two hours. Micro Bio-Spin 6 chromatography columns (Bio-Rad Laboratories, Hercules, CA, USA) were used for purification of the labeled RNA. After drying the samples completely, samples were prepared for hybridization by adding nuclease-free water, Hyb Spike-In solution, 10X GE Blocking Agent and 2X Hi-RPM Hybridization Buffer. Samples were hybridized to a custom-designed 8x60k Agilent microarray. The custom microarray covered all murine and canine miRNAs from miRBASE version 17.0 [103] and contained probes for 1386 individual miRNAs with each miRNA covered by 40 different features. Hybridizations were performed in hybridization chambers (Agilent) for 20 hours at 55°C and the slides were washed as described in the protocol. Arrays were scanned at a resolution of 2 μm using an Agilent G2505C Scanner. The microarray data discussed in this publication have been deposited in NCBIs Gene Expression Omnibus (GEO, http://www.ncbi.nlm.nih.gov/geo/) and are accessible through GEO Series accession number GSE36976.

### Microarray data analysis

Feature Extraction Software Version 10.7.3.1. was used for grid alignment and data extraction. Signal intensities for each feature were scanned and calculated by subtracting the background. The flag “is well above background” (WABG) produced by the Feature Extraction Software was used to filter signals above background noise. Features had to contain a minimum of two WABG flags “1” in at least one of the experimental groups (detectable in at least two samples of the control or the DCM group) to be included in the further analysis. Mean signal intensities were calculated for each detectable miRNA probe and subsequently normalized between samples using the BioConductor package “vsn” [104]. For quality control, normalized data were analyzed with a heatmap based on pair-wise distances (BioConductor package geneplotter). Software „LIMMA“(Bioconductor) [105] was used for significance analysis (function “decideTests”) and assessment of miRNA expression changes.

### Quantitative real-time RT-PCR (qPCR)

After total RNA purification, reverse transcription was performed using the miScript Reverse Transcription Kit (Qiagen) according to manufacturer′s protocol. The miScript SYBR Green PCR Kit (Qiagen) was used for qPCR according to the manufacturer′s protocol. The miScript Primer Assays *cf_miR-92a_1, mm_miR-142-3p_1*, *cf_let-7c_1*, mm_miR_144*_1 and *cf_miR-21_1* were used as target miRNAs. hs_RNU6B_2 and hs_RNU1A_1 were used as endogenous control assays*.* All samples were run as duplicates. To account for spurious PCR amplification of contaminating genomic DNA, a control containing total RNA without reverse transcription was included. As negative controls, the amplification mixture of each assay was run without adding the reverse transcribed RNA. Relative quantification was carried out using the Δ Δ threshold cycle (Ct) method with hs_RNU6B_2 and hs_RNU1A_1 as endogenous controls. The TaqMan 7500 Real Time PCR System (Applied Biosystems, Life Technologies Corporation, Carlsbald, CA, USA) was used for qPCR.

### Statistical analysis

#### Study population

The software GraphPad Prism version 5.04 for Windows (GraphPad Software, La Jolla, CA) was used for statistical analysis of the cohort. Mann–Whitney one-way test was used to calculate differences for all parameters in Table 1.

#### miRNA microarray

A “moderated *t*-test” (Software “LIMMA”) was used for statistical analysis of the mean miRNA expression differences in the miRNA microarray. Multiple testing correction was performed using the parameter “FDR” (false discovery rate) in the function “decideTests” for adjustment of p-values. A p-value < 0.05 was considered to be statistically significant. FC was calculated (Software “LIMMA”) and included into the assessment of expression ratios (FC at least 1.5-fold was considered to be significant). Software program Multi Experiment Viewer (MeV v.4.7.1) was used for hierarchical clustering of 22 different miRNAs using HCL support trees, Pearson correlation and 200 iterations.

#### qPCR

Target assays were normalized to internal controls hs_RNU6B_2 and hs_RNU1A_1 which had been found to be suitable as controls in dog serum in a preliminary test*.* The software “REST 2009” was used for analysis of relative expression in PCR assays [106,107]. Mean values of twice prepared approaches were used. A p-value < 0.05 was considered to be statistically significant.

## Abbreviations

BSA: Body surface area; Ct: Threshold cycle; cTnI: Cardiac Troponin I; DCM: Dilated cardiomyopathy; ECG: Electrocardiogram; EF: Ejection fraction; FC: Fold change; FDR: False discovery rate; Holter: 24-hour ambulatory electrocardiogram; ICM: Ischemic cardiomyopathy; LA/Ao: Ratio of the left atrium to the aorta; LVEDV: Left ventricular end-diastolic volume; LVESV: Left ventricular end-systolic volume; miRNA: MicroRNA; qPCR: Real-Time RT-PCR; VPC(s): Ventricular premature contraction(s); WABG: Well above background.

## Competing interest

The authors declare that they have no competing interests.

## Authors’ contributions

CS, KW and GW designed the study. GW carried out and CS participated in all patient examinations, classified groups and collected material. CS and KW carried out all laboratory procedures. SB and KW designed the miRNA microarray, SB participated in the microarray procedure, analyzed data and performed statistical analysis. CS drafted the manuscript. KW, GW and SB helped to enhance the manuscript. All authors read and approved the final manuscript.

## Authors’ information

Carola Steudemann (CS), DVM, Clinic of Small Animal, LMU University of Munich

Dr. rer. nat. Stefan Bauersachs (SB), team leader, Functional Genomics of Reproduction, Genomics Unit, Laboratory of Functional Genome Analysis, Gene Center, LMU University of Munich

Dr. med. vet. Karin Weber (KW), DVM, specialist for veterinary physiology, head of laboratory diagnostics and molecular biology, Clinic of Small Animal, LMU University of Munich

PD, Dr. med. vet., Dr. vet. med. habil. Gerhard Wess (GW), DVM, Dipl. ACVIM (Cardiology), Dipl. ECVIM-CA (Cardiology and Internal Medicine), head of cardiology service, Clinic of Small Animal, LMU University of Munich.

## Availability of Supporting Data

Supporting data has been deposited in NCBIs Gene Expression Omnibus (GEO, http://www.ncbi.nlm.nih.gov/geo/) and is accessible through GEO Series accession number GSE36976.
